# Prevalence and Genotype Distribution of Human Papillomavirus Among Males in Shantou, China (2019–2023)

**DOI:** 10.1002/hsr2.71094

**Published:** 2025-07-18

**Authors:** Chusheng Huang, Tongtong Xiao, Fan Yang, Xiaoxia Ma

**Affiliations:** ^1^ Department of General Surgery Shantou Central Hospital Shantou Guangdong China; ^2^ Department of Clinical Laboratory Shantou Central Hospital Shantou Guangdong China

**Keywords:** China, epidemiology of HPV in males, HPV genotyping, HPV prevalence, human papillomavirus, Shantou

## Abstract

**Background and Aims:**

Human papillomavirus (HPV) is one of the most prevalent sexually transmitted infections globally, posing significant public health challenges. However, research in China has largely focused on female populations, with limited data on male HPV prevalence and genotype distribution. This study aims to evaluate HPV prevalence rates and analyze the distribution of various HPV genotypes among male participants in Shantou, China.

**Methods:**

A retrospective study was conducted, analyzing data from 2399 male patients who underwent HPV screening at Shantou Central Hospital between 2019 and 2023. PCR‐reverse dot blot hybridization techniques were employed to identify and characterize 21 distinct HPV genotypes in the study population.

**Results:**

The overall HPV positivity rate was 44.27% (1062/2399; 95% CI: 42.28%–46.26%), with high‐risk HPV (HR‐HPV) accounting for 24.47% (587/2399; 95% CI: 22.75%–26.19%) and low‐risk HPV (LR‐HPV) for 32.06% (769/2399; 95% CI: 30.19%–33.92%). Single infections were more common (27.64%), with HPV16 (5.21%), HPV52 (5.13%), and HPV51 (4.54%) being the most prevalent HR‐HPV genotypes. HPV6 (18.51%) and HPV11 (10.38%) were the most common LR‐HPV types. Coinfections involving HPV6, HPV11, and HPV16 were frequently observed. The nine‐valent HPV vaccine (9vHPV) covered 65.25% of LR‐HPV and 42.00% of HR‐HPV types in this study.

**Conclusion:**

Our findings underscore the high burden of HPV infections among males in Shantou, highlighting the need for targeted prevention strategies, including vaccination programs, to reduce the incidence of HPV‐related diseases in this population.

## 1 Introduction

Human papillomavirus (HPV) is a widespread sexually transmitted virus that can also be transmitted through nonsexual routes, including fomites, skin contact, and vertical transmission from mother to child [1, 2, 3]. Globally, HPV prevalence among males varies considerably by region, ranging from 1% to over 40%, influenced by factors such as population demographics, sexual behavior, and healthcare access [4]. A recent meta‐analysis reported HPV prevalence rates of 52.45% among Chinese male outpatients and 7.89% in individuals undergoing routine health examinations [5]. HPV is a key etiological agent in various male cancers, including anal, penile, and oropharyngeal malignancies [6]. Routine HPV testing and screening in males are essential for early detection and prevention of HPV‐related diseases.

HPV comprises over 200 genotypes [7], classified into high‐risk (HR) and low‐risk (LR) types based on their oncogenic potential [8]. HR‐HPV genotypes, including HPV 16, 18, 31, 33, 35, 39, 45, 51, 52, 56, 58, 59, 66, 68, 83, and others, are closely associated with cancer development [9, 10]. In the realm of these pathological phenomena, the viral oncogenes E1‐E7 assume a crucial role. Specifically, E6 promotes the degradation of the tumor suppressor protein p53 by binding to it, while E7 disrupts the function of the retinoblastoma protein, resulting in the misregulation of cellular proliferation [11]. LR‐HPV genotypes, including HPV 6, 11, 40, 42, 43, 44, 61, 70, 72, 81, and others, cause benign and localized lesions [12]. In men, HPV also acts as a viral reservoir, enabling transmission to female partners and sustaining population‐level prevalence. Despite its clear relevance to male health, most research on HPV has historically concentrated on women, largely due to the high global burden of cervical cancer [13, 14, 15].

Currently available HPV vaccines—particularly the 9‐valent vaccine—have demonstrated robust efficacy and safety profiles. However, accessibility and cost remain substantial barriers in many regions [16]. In mainland China, HPV vaccines are not yet approved for use in males, highlighting the need for a better understanding of the epidemiological burden in this population to support future public health strategies. Identifying population‐specific HPV patterns is vital for tailoring prevention strategies and optimizing vaccine deployment.

Although HPV research in China has primarily focused on females, data on HPV prevalence and genotype distribution among males remain limited [17]. In particular, specific epidemiological data for Shantou, a densely populated medical hub in eastern Guangdong Province, are lacking. Due to its unique demographic and socioeconomic characteristics, the male population in Shantou may exhibit distinct HPV prevalence and genotype patterns compared to other regions. Understanding these local trends is critical for the development of targeted HPV prevention initiatives, including vaccination and screening programs tailored to the region's specific needs.

This study aims to assess the prevalence of HPV infections and analyze the genotype distribution among males in Shantou, China, from 2019 to 2023. By focusing on this population, we aim to fill the current research gap and provide insights that could shape public health policies, especially regarding HPV vaccination for males.

## 2 Materials and Methods

### 2.1 Study Design and Patients

This retrospective study was conducted at Shantou Central Hospital and covered the period from January 1, 2019 to December 31, 2023. The study population consisted of male patients who underwent HPV screening for various reasons, including routine physical examinations, patient‐initiated requests, physician‐recommended diagnostic evaluations, and opportunistic screenings. Specific indications for testing included general health check‐ups, urinary tract infections (UTIs), genital warts, condyloma, balanitis, penile neoplasms, and eczema. Our investigation included 2399 male participants who met the following eligibility requirements: (1) male sex; (2) availability of complete clinical data; (3) willingness to provide adequate genital specimens for HPV genotyping; and (4) absence of any previous HPV therapeutic interventions or application of medications to the reproductive tract during the 7 days prior to specimen collection.

### 2.2 Specimen Collection

Samples were collected by gently swabbing the penile squamous regions, including the shaft and glans, using saline‐soaked nylon‐tipped swabs. Following collection, these swabs were immediately immersed in 1 mL of physiological saline and maintained at refrigeration temperature (4°C) pending laboratory processing and examination.

### 2.3 HPV DNA Amplification and Genotyping

HPV genotyping was conducted using the 21 HPV GenoArray Diagnostic Kit (Kaipu Biotechnology, China), which utilizes DNA amplification targeting the HPV L1 gene along with a flow‐through hybridization method. This kit has been authorized by the State Food and Drug Administration (Permit Number: 20143402188). It is capable of detecting 15 high‐risk HPV (HR‐HPV) types (HPV 16, 18, 31, 33, 35, 39, 45, 51, 52, 53, 56, 58, 59, 66, and 68) and 6 low‐risk HPV (LR‐HPV) types (HPV 6, 11, 42, 43, 44, and CP8304). All protocols were performed according to the supplier's manual. The process was as follows: (1) DNA extraction: DNA was extracted using a magnetic protein denaturant within a magnetic bead binding solution, which dissolves the protein and separates the DNA. The released DNA then binds to the magnetic bead, and impurities are removed using a washing solution. The purified DNA is eluted with an eluent. This process was performed using an automated nucleic acid extraction device with a pre‐packed nucleic acid extraction kit (DaAn Gene Co. Ltd., China). (2) PCR amplification: The extracted DNA underwent PCR amplification with HPV L1 consensus primers. The amplification reagent mixture consisted of 23.25 μL of PCR mixture, 0.75 μL of DNA polymerase, and 1 μL of DNA/sample. The amplification protocol began with initial denaturation at 20°C for 10 min, followed by 9 min at 95°C, and 40 cycles consisting of 20 s at 95°C, 30 s at 55°C, and 30 s at 72°C, ending with a final 5‐min extension at 72°C. (3) Hybridization: A volume of 25 μL of the PCR products was examined using flow‐through hybridization with the HPV GenoArray Diagnostic Kit. The specific steps were as follows: 25 μL of PCR products were denatured at 95°C for 5 min, followed by cooling on ice for 2 min. The samples were mixed with 0.5 mL of prewarmed hybridization solution and incubated for 10 min, after which a blocking solution was added. The flow‐through hybridization took place in a sample well above a Hybrimem HPV‐21/HPV‐37 membrane containing immobilized probes targeting the HPV DNA. Streptavidin–horseradish peroxidase conjugate was added to bind to the biotinylated PCR products. Positive results were visually detected through the purple dot produced by the breakdown of the substrate nitroblue tetrazolium‐5‐bromo‐4‐chloro‐3‐indolylphosphate. These results were compared with the membrane's schematic to identify the corresponding HPV DNA type. Both HPV negative and positive controls provided in the kit were tested simultaneously to ensure quality control.

### 2.4 Statistical Analysis

Data analysis was conducted using Microsoft Excel 2021. The *χ* ^2^ test was applied to compare the sample rates between the groups. For calculating the *χ* ^2^ values, *p* values, and 95% confidence intervals (95% CI), SPSS 20 (SPSS Inc., Chicago, IL, USA) was utilized. A two‐tailed *p* < 0.05 was regarded as statistically significant. Graphical presentations were created using R 4.2.3.

## 3 Results

### 3.1 The Overall HPV Infection Rate

**Figure 1:**
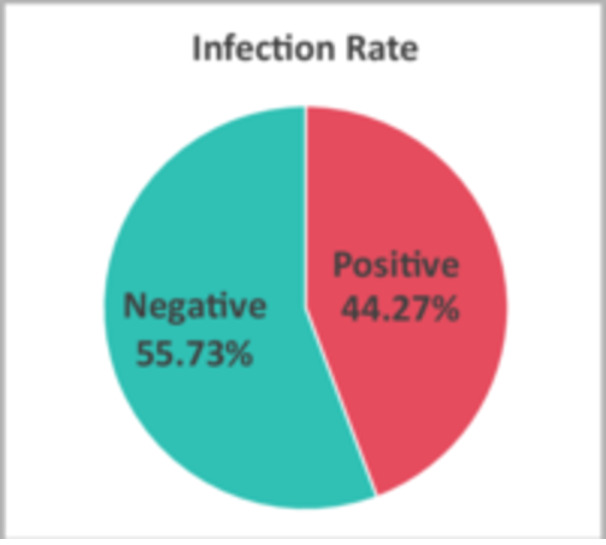
Human papillomavirus infection rates in 2399 cases of patients.

A total of 2399 participants were included, with a mean age of 32.83 ± 9.41 years (range: 5–76 years). The overall HPV positivity rate was 44.27% (1062/2399; 95% CI: 42.28%–46.26%) [Figure 1]. HR‐HPV accounted for 24.47% (587/2399; 95% CI: 22.75%–26.19%), while LR‐HPV was observed in 32.06% (769/2399; 95% CI: 30.19%–33.92%). Most HPV‐positive patients exhibited symptoms such as warts, dermatitis, acroposthitis, urethritis, rash, and prostatitis. Among the patients, 26.27% were asymptomatic, while warts were the most common diagnosis, accounting for 50.94%, surpassing other diagnostic types (Table 1).

**Table 1:** The distribution of different diagnosis types in 1062 HPV‐positive males.

Diagnosis	Number	Frequency (%)
Wart	541	50.94
Dermatitis	71	6.69
Acroposthitis	17	1.60
Rash	55	5.18
Urethritis	15	1.41
Prostatitis	20	1.88
Others	64	6.03
Physical examination	279	26.27

**Figure 2:**
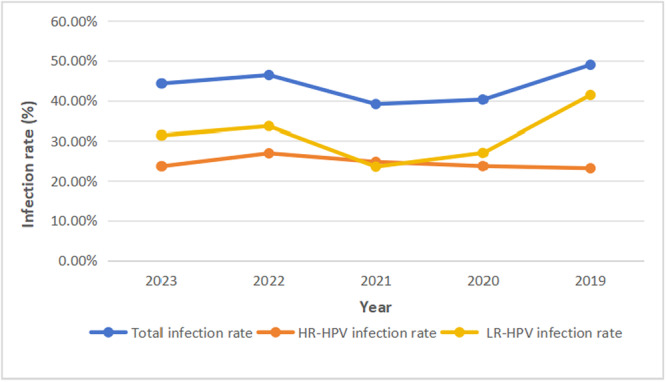
Trends in the overall prevalence of HPV infection, high‐risk HPV infection, and low‐risk HPV infection from 2019 to 2023.

As shown in Figure 2, the overall positive detection rate ranged from 38.08% to 48.96% between 2019 and 2023. The HPV infection rates varied significantly across different years (*χ* ^2^ = 12.581, df = 4, *p* < 0.05). The highest overall HPV infection rate was observed in 2019 (48.96%), while the lowest rate was in 2021 (39.08%). No significant differences were found in the rates of HR‐HPV infection from 2019 to 2023 (*χ* ^2^ = 2.559, df = 4, *p* = 0.63). However, the infection rates of LR‐HPV differed significantly across the years (*χ* ^2^ = 39.645, df = 4, *p* < 0.001).

The single infection rate was 27.64% (663/2399), while the multiple infection rate was 16.63% (399/2399), indicating that single infection was the predominant pattern of HPV infection among men. Among the 399 men with multiple infections, the prevalence of HR‐HPV and HR‐HPV coinfection, HR‐HPV and LR‐HPV coinfection, and LR‐HPV and LR‐HPV coinfection was 2.63% (63 cases), 12.26% (294 cases), and 1.75% (42 cases), respectively. Therefore, HR‐HPV and LR‐HPV coinfection was the most common, followed by HR‐HPV and HR‐HPV coinfection, with LR‐HPV and LR‐HPV coinfection being the least common. Further analysis showed that double infections had a prevalence of 10.00%, followed by triple infections (4.04%) and other multiple infections (2.58%). Among the 633 men with single infections, the detection rates of HR types and LR types were 9.59% (230/2399) and 18.05% (433/2399), respectively, indicating that single LR‐HPV was the predominant type of infection among positive cases (Table 2).

**Table 2:** Prevalence of infection patterns and infection types of 21 genotypes from 2019 to 2023.

	Positive cases	% (95% CI)	2019 (*n* = 527)	2020 (*n* = 363)	2021 (*n* = 412)	2022 (*n* = 539)	2023 (*n* = 558)
Positive cases	1062	44.27 (42.28–46.26)	258 (48.96%)	146 (40.22%)	161 (39.08%)	250 (46.38%)	247 (44.27%)
Infection types							
Single L	433	18.05 (16.51–19.59)	122 (23.15%)	58 (15.98%)	55 (13.35%)	93 (17.25%)	105 (18.82%)
Single H	230	9.59 (8.41–10.77)	31 (5.88%)	41 (11.29%)	49 (11.89%)	55 (10.20%)	54 (9.68%)
LR + LR	42	1.75 (1.23–2.28)	14 (2.66%)	2 (0.55%)	4 (0.97%)	12 (2.23%)	10 (1.79%)
HR + HR	63	2.63 (1.99–3.27)	9 (1.71%)	7 (1.93%)	15 (3.64%)	14 (2.6%)	18 (3.23%)
LR + HR	294	12.26 (10.94–13.57)	82 (15.56%)	38 (10.47%)	38 (9.22%)	76 (14.10%)	60 (10.75%)
Infection pattern							
1	663	27.64 (25.85–29.43)	153 (29.03%)	99 (27.27%)	104 (25.24%)	148 (27.46%)	159 (28.49%)
2	240	10.00 (8.80–11.20)	58 (11.01%)	31 (18.54%)	46 (11.17%)	46 (8.53%)	59 (10.57%)
3	97	4.04 (3.26–4.83)	31 (5.88%)	8 (2.20%)	5 (1.21%)	30 (5.57%)	23 (4.12%)
≥ 4	62	2.58 (1.95–3.22)	16 (3.04%)	8 (2.20%)	6 (1.46%)	26 (4.82%)	6 (1.08%)
≥ 2	399	16.63 (15.14–18.12)	105 (19.92%)	47 (12.95%)	57 (13.83%)	102 (18.92%)	88 (15.77%)

### 3.2 HPV Genotype Distribution

**Figure 3:**
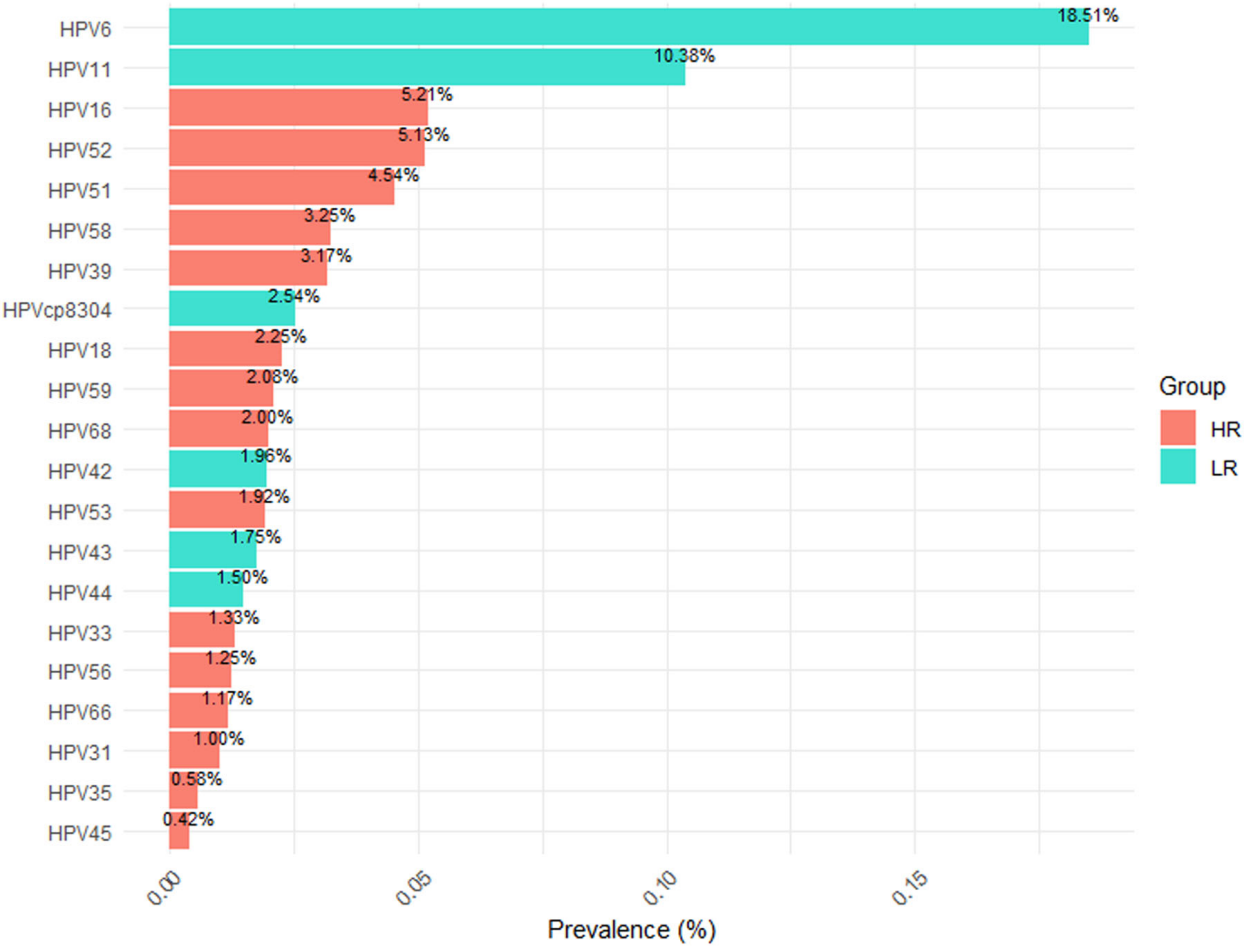
The prevalence and genotype distribution of HPV (HR‐HPV and LR‐HPV) infections among males from 2019 to 2023.

From January 2019 to December 2023, the six most common HR‐HPV genotypes were HPV 16, 52, 51, 58, 39, and 18, with overall frequencies of 5.21%, 5.13%, 4.54%, 3.25%, 3.17%, and 2.25%, respectively, as shown in Figure 3. These were followed by HPV 59, 68, 53, 33, 56, 66, 31, 35, and 45. Additionally, the most common LR‐HPV genotypes were HPV6 (18.51%), HPV11 (10.38%), and HPVCP8304 (2.54%).

**Figure 4:**
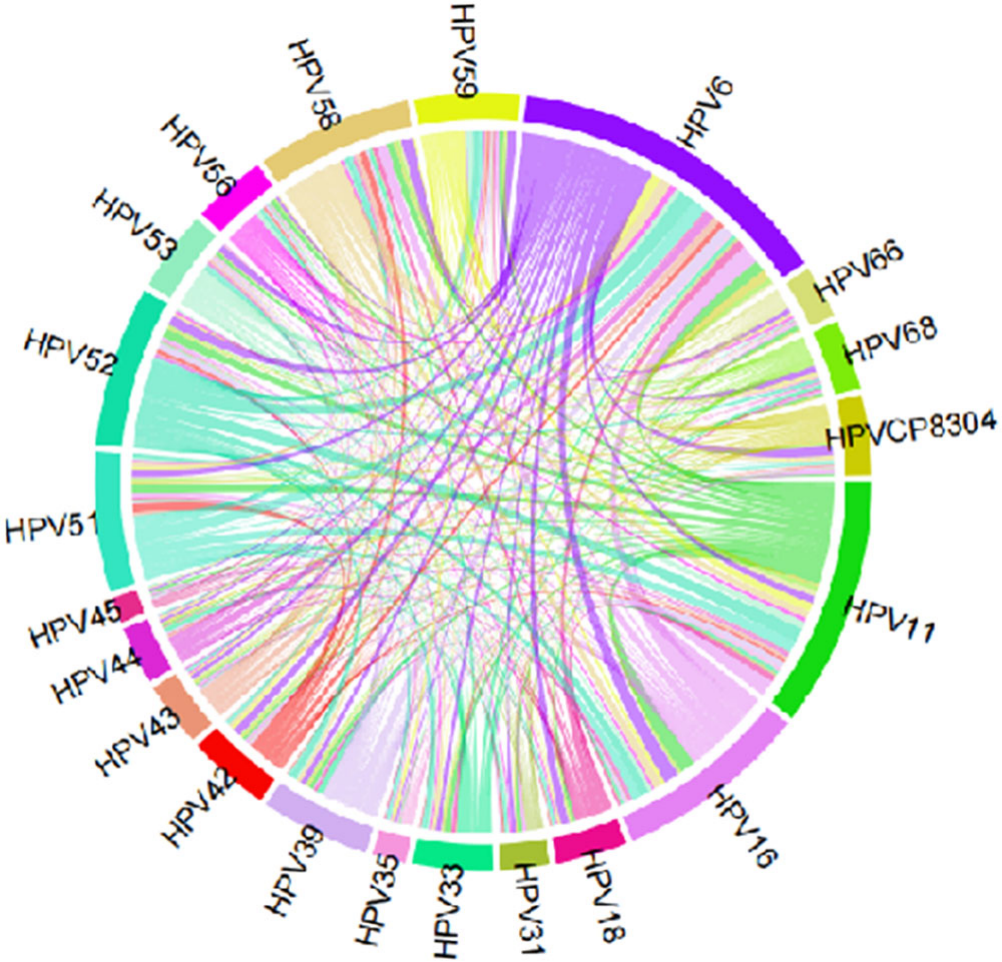
Chord diagram of HPV type correlations in males with multiple infections in Shantou, China.

The chord diagram illustrates various combinations of HPV types in coinfections, with the most frequent combinations being HPV6 and HPV16 (36 occurrences), HPV11 and HPV16 (34 occurrences), HPV6 and HPV52 (29 occurrences), HPV11 and HPV52 (26 occurrences), and HPV6 and HPV56 (24 occurrences). Coinfections involving HPV6, HPV11, and HPV16 were notably frequent, as shown in Figure 4.

### 3.3 Analysis of the Prevalence of 9‐Valent HPV Vaccine Targeting Genotypes HPV 6, 16, 18, 31, 33, 45, 52, and 58

**Figure 5:**
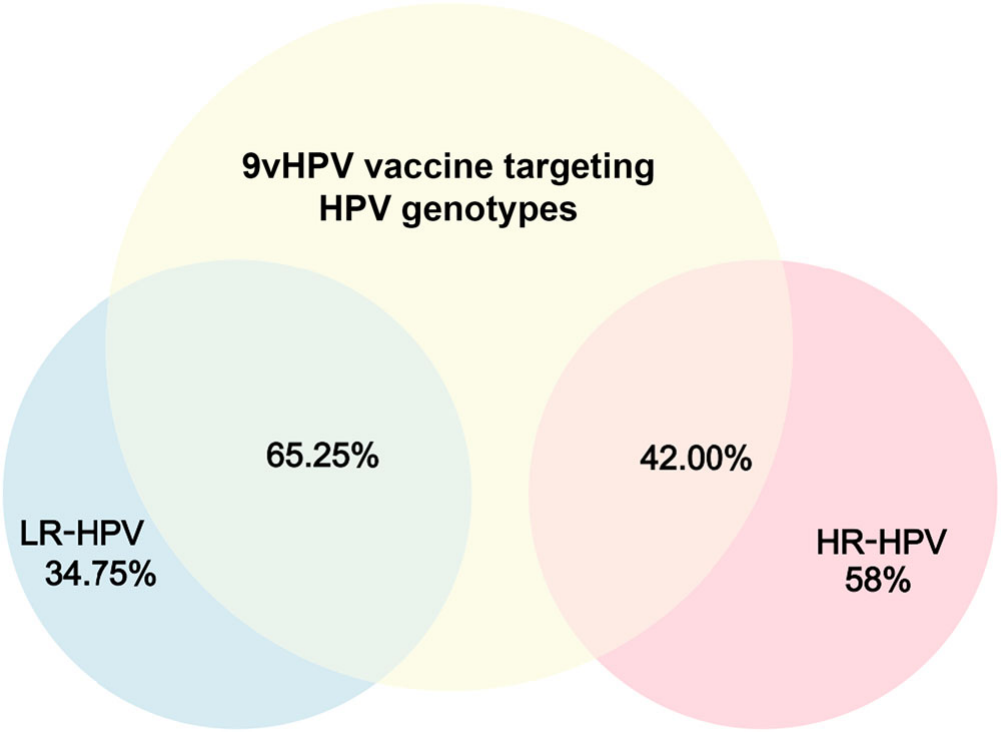
The prevalence of 9v human papillomavirus (HPV) vaccine targeting genotypes among 1062 males.

The currently approved nine‐valent HPV (9vHPV) vaccine provides the most comprehensive protection available, targeting two LR‐HPV types (HPV 6 and 11) and seven HR‐HPV types (HPV 16, 18, 31, 33, 45, 52, and 58). An analysis of genotype prevalence targeted by the 9vHPV vaccine is presented in Figure 5, showing that 65.25% of LR‐HPV types and 42.00% of HR‐HPV types are preventable by the vaccine. Detailed prevalence data for these nine targeted HPV genotypes are provided in Table 3. In addition to HPV16 (11.77%) and HPV52 (11.58%), three other vaccine‐targeted genotypes—HPV 33 (3.01%), HPV 31 (2.26%), and HPV 45 (0.94%)—were found to have notably low prevalence. Importantly, 58.00% of high‐risk HPV genotypes (such as HPV51, HPV 39, HPV59, HPV 68, and HPV 53) fall outside the protective scope of the 9vHPV vaccine.

**Table 3:** Distribution of HPV genotypes in 1062 HPV positive men.

	Number	% (95% CI)
HR‐HPV types		
HPV16	125	11.77 (9.83–13.71)
HPV52	123	11.58 (9.66–13.51)
HPV51	109	10.26 (8.44–12.09)
HPV58	78	7.34 (5.76–8.91)
HPV39	76	7.16 (5.61–8.71)
HPV18	54	5.08 (3.76–6.41)
HPV59	50	4.71 (3.43–5.98)
HPV68	48	4.52 (3.27–5.77)
HPV53	46	4.33 (3.11–5.56)
HPV33	32	3.01 (1.99–4.04)
HPV56	30	2.82 (1.83–3.82)
HPV66	28	2.64 (1.67–3.60)
HPV31	24	2.26 (1.37–3.15)
HPV35	14	1.32 (0.63–2.00)
HPV45	10	0.94 (0.36–1.52)
LR‐HPV types		
HPV6	444	41.81 (38.84–44.77)
HPV11	249	23.45 (20.90–25.99)
HPVCP8304	61	5.74 (4.34–7.14)
42	47	4.43 (3.19–5.66)
43	42	3.95 (2.78–5.13)
44	36	3.39 (2.30–4.48)

### 3.4 HPV Genotype Infection in Different Age Groups

**Figure 6:**
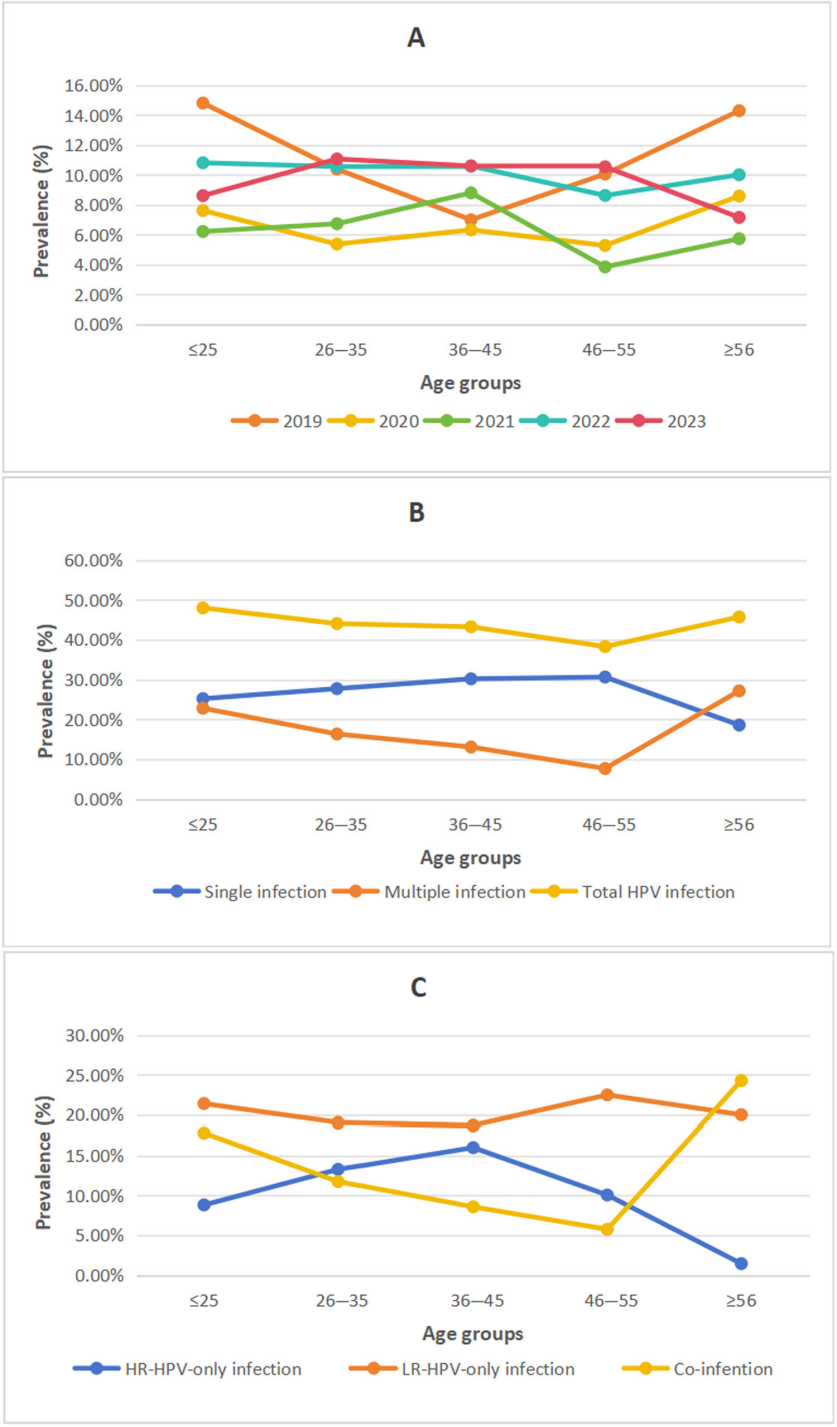
The prevalence of HPV infection by age groups. (A) HPV infections from 2019 to 2023. (B) Single infection, multiple infection, and total infection. (C) Infection with high‐risk‐HPV only, infection with low‐risk‐HPV only and coinfection.

The HPV infection rates for the age groups ≤ 25, 26–35, 36–45, 46–55, and ≥ 56 years were 48.00% (240/500), 44.05% (518/1176), 43.24% (192/444), 38.28% (80/209), and 45.71% (32/70), respectively. As shown in Table 4, the highest prevalence was observed in the ≤ 25‐year group at 48.00%, followed by the ≥ 56‐year group at 45.71%, while the lowest prevalence was recorded in the 46–55‐year group at 38.28% (Figure 6A). Notably, the infection rates in the ≤ 25 and ≥ 56 age groups were higher than the overall prevalence (44.27%). The prevalence of single HPV infection was highest in the 46–55‐year group at 30.62%, whereas multiple HPV infection peaked in the ≥ 56‐year group at 30.00%. Single infections predominated in all age groups except in the ≥ 56‐year group (Table 4 and Figure 6B). In terms of infection type, all age groups, except for the ≥ 56‐year group, were predominantly characterized by LR‐HPV‐only infections (Table 4 and Figure 6C).

**Table 4:** Prevalence of HPV infection at different age groups among 2399 males from 2019 to 2023.

	Positive cases	% (95% CI) for all samples	≤ 25 (*n* = 500)	26–35 (*n* = 1176)	36–45 (*n* = 444)	46–55 (*n* = 209)	≥ 56 (*n* = 70)
Positive cases	1062	44.27 (42.28–46.26)	240 (48.00%)	518 (44.05%)	192 (43.24%)	80 (38.28%)	32 (45.71%)
Year							
2019	258	10.75 (9.51–11.99)	74 (14.80%)	122 (10.37%)	31 (6.98%)	21 (10.05%)	10 (14.29%)
2020	146	6.09 (5.13–7.04)	38 (7.60%)	63 (5.36%)	28 (6.31%)	11 (5.26%)	6 (8.57%)
2021	161	6.71 (5.71–7.71)	31 (6.20%)	79 (6.72%)	39 (8.78%)	8 (3.83%)	4 (5.71%)
2022	250	10.42 (9.20–11.64)	54 (10.80%)	124 (10.54%)	47 (10.59%)	18 (8.61%)	7 (10.00%)
2023	247	10.30 (9.08–11.51)	43 (8.60%)	130 (11.05%)	47 (10.59%)	22 (10.53%)	5 (7.14%)
Infection pattern							
Single	663	27.64 (25.85–29.43)	127 (25.40%)	325 (27.64%)	134 (30.18%)	64 (30.62%)	13 (18.57%)
Multiple	399	16.63 (15.14–18.12)	120 (24.00%)	177 (15.05%)	62 (13.96%)	19 (9.09%)	21 (30.00%)
Infection types							
HR only	293	12.21 (10.90–13.52)	44 (8.80%)	156 (13.27%)	71 (15.99%)	21 (10.05%)	1 (1.43%)
LR only	475	19.80 (18.21–21.39)	107 (21.40%)	224 (19.05%)	83 (18.69%)	47 (22.49%)	14 (20.00%)
HR + LR	294	12.26 (10.92–13.57)	89 (17.80%)	138 (11.73%)	38 (8.56%)	12 (5.74%)	17 (24.29%)

**Figure 7:**
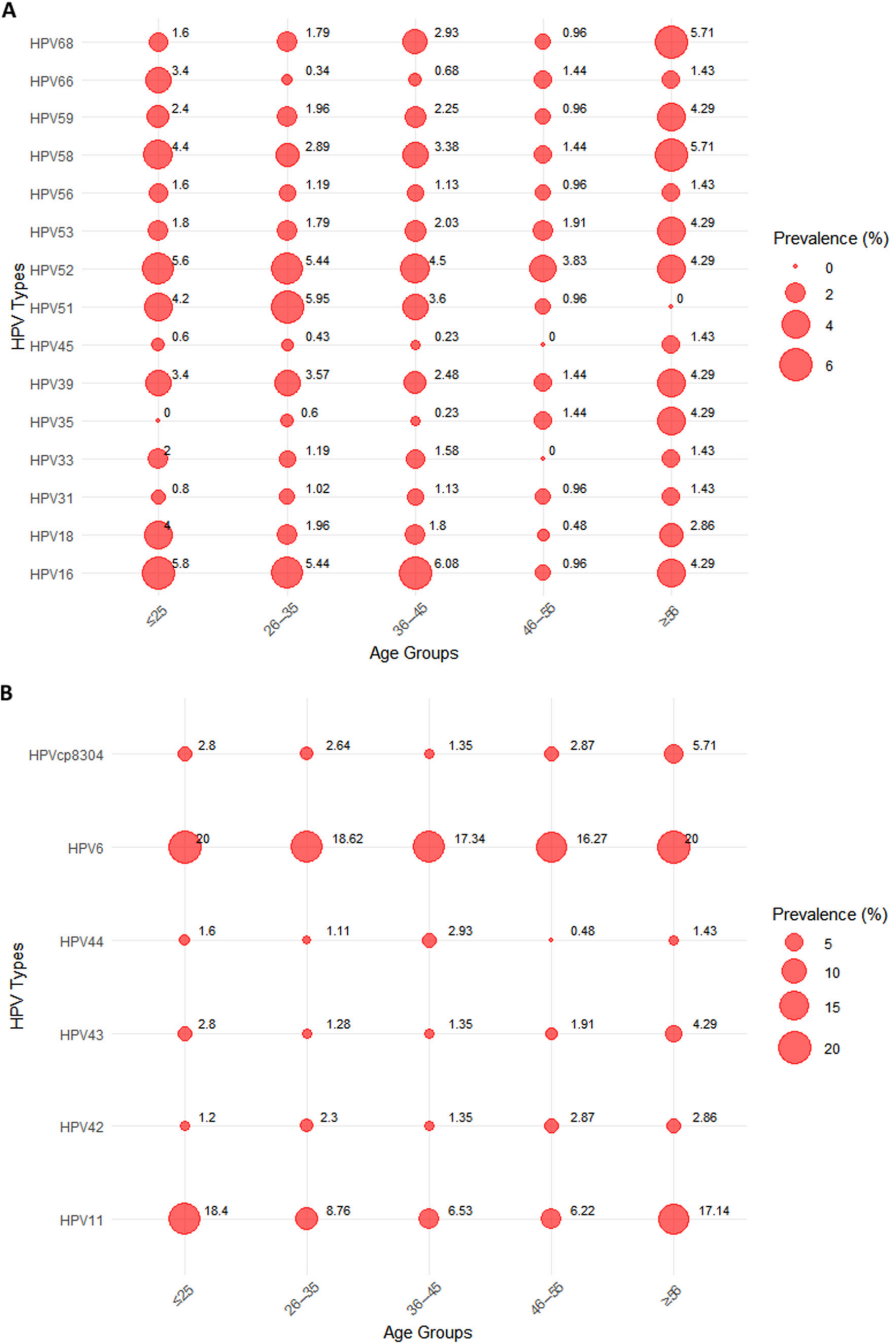
Bubble plots showing the relative prevalence of detected 21 HPV genotypes across age groups. (A) 15 high‐risk HPV genotypes. (B) 6 low‐risk HPV genotypes.

The distribution of HR‐HPV types varied significantly across age groups. In younger age groups (≤ 25 and 26–35 years), HPV16 and HPV52 exhibited the highest prevalence rates, with infection rates of up to 5.8% and 5.6%, respectively. In contrast, older age groups, particularly the ≥ 56‐year group, showed an elevated prevalence of HPV58 and HPV68, each reaching 5.71% (Figure 7A). Across all age groups, LR‐HPV infections were predominantly driven by HPV6 and HPV11. These two types consistently demonstrated the highest infection rates, particularly in the ≤ 25 and ≥ 56 age groups, where HPV6 reached a prevalence of 20%, and HPV11 showed prevalence rates of 18.4% and 17.14%, respectively. Other LR‐HPV types, such as HPV42, HPV43, HPV44, and HPVCP8304, exhibited relatively low and evenly distributed infection rates across age groups, except for HPVCP8304, which reached 5.71% in the ≥ 56‐year group (Figure 7B).

## 4 Disccusion

HPV is a sexually transmitted virus with a high prevalence among men. Characterizing HPV infection and genotype distribution in men is a critical clinical issue, particularly for preventing genital cancer in men and, consequently, reducing HPV infection in women. Despite the importance, most studies on HPV infection in China have focused on women, and data on the epidemiology of HPV infection in men are quite limited.

This study represents the first investigation into the prevalence of male HPV infection in the Shantou region, providing significant epidemiological insights. From 2019 to 2023, the overall prevalence of male HPV infection in Shantou was found to be 44.27%, reflecting the regional burden of HPV among men. In the context of global literature, this prevalence falls within the mid‐range of reported rates worldwide. A 2023 systematic review estimated a global male HPV prevalence of ~31%, with lower rates observed in Eastern and Southeastern Asia [4]. The prevalence in Shantou (44.27%) exceeds this global average but aligns with large‐scale studies in North America, such as one from the United States (46.9%) [18]. However, Shantou's prevalence is notably lower than figures reported in certain high‐risk populations, such as the Czech Republic (96.8% among predominantly HIV‐positive MSM) [19], Brazil (80% among partners of women with cervical intraepithelial neoplasia) [20], and Hong Kong (90% in MSM populations) [21]. These disparities likely stem from differences in study populations, sampling methodologies, and risk factor profiles rather than actual geographical variations in HPV burden. When compared specifically with other Asian regions, our findings suggest that Shantou may have a higher male HPV prevalence than the Eastern and Southeastern Asian average reported in the global review. This observation highlights potential unique regional risk factors or methodological differences that merit further investigation.

In China, the epidemiological trends of male HPV infection demonstrate substantial variability. A 2018 meta‐analysis revealed a high prevalence of HPV infection among Chinese men, with an overall infection rate of 14.5% among heterosexual men and 59.9% among men who have sex with men (MSM) [17]. Comparisons within China indicate that the prevalence of HPV infection in Shantou (44.27%) is similar to the 42.15% prevalence reported in Guangzhou [22] but is lower than the rates reported in Qingyuan [23] (54.3%), Liaocheng [24] (64.87%), and Shanghai [25] (65.5%). Conversely, it is higher than the rates observed in Yunnan [26] (23.8%), Chongqing [27] (14.9%), and Liuzhou [28] (9.4%). These discrepancies may be attributed to various factors, such as demographic factors, patterns of sexual behavior, levels of health literacy, and public health initiatives. Demographic attributes, such as age distribution, gender ratios, and socioeconomic conditions, vary across regions and can influence HPV prevalence. Sexual behavior patterns, including the number of sexual partners and the use of safe sex practices, significantly impact the risk of HPV transmission, particularly among sexually active populations where the risk is heightened [29, 30].

The genotype distribution of HPV infection in males varies across countries. Between 1995 and 2022, the five most prevalent HR‐HPV types worldwide were HPV16, HPV51, HPV52, HPV59, and HPV18 [6]. Consistent with our findings, HPV16 was the most prevalent HR‐HPV type in Italy [31], Vietnam [32], and Japan [33]. However, notable differences were observed in studies conducted in other regions, such as Guangzhou (HPV52, 16, and 51) [7], Chongqing (HPV52, 16, and 58) [12], and Liuzhou (HPV58, 52, and 39)^[13]^ in China, as well as Brazil (HPV89, 62, and 61) [34], the United States (HPV64, 82, and 89) [35], and Tanzania (HPV52, 51, and 16) [36]. These discrepancies in the distribution of HPV genotypes across regions may be attributed to regional preferences for HPV assays with varying sensitivities in detecting specific genotypes [37].

Among LR‐HPV genotypes, HPV6 (18.51%) and HPV11 (10.38%) were the most common, which is consistent with findings from other studies [38, 39, 40]. HPV6 and HPV11 are commonly associated with the development of condylomatous lesions and are responsible for ~90% of all external genital warts [41]. The high positivity rates of HPV6 and HPV11 in our study could be due to the fact that most male patients presented with warts, which are frequently linked to these genotypes.

Among the 1062 HPV‐infected subjects, single HPV‐type infections were predominant, accounting for 62.43%, compared to 37.57% for multiple infections. The prevalence of mixed infections involving two or more HPV types decreased as the number of HPV types increased. Studies have shown that the presence of multiple HPV genotypes may prolong the duration of infection and increase the risk of cancer [42]. Multiple HR‐HPV infections in men have been linked to increased oxidative stress, inflammation, and altered sperm parameters, potentially contributing to male infertility [43]. Therefore, multiple infections are considered more dangerous than single infections. In this study, multiple HPV infections were predominantly observed in individuals below the age of 25 and above 56, suggesting that an active and potentially unregulated sexual lifestyle may contribute to increased infection risk among younger individuals. For older men, the increase in multiple HPV infections is likely due to an age‐related decline in immune function, making them more susceptible to persistent infections. However, other studies have also indicated that there may be competition or a counterbalance between different HPV types [44]. Infection with multiple HPV subtypes increases the risk of abnormal cell proliferation and malignant transformation [45]. Therefore, it is crucial to mitigate the risk of carcinogenesis in infected tissues by increasing the frequency of follow‐ups, conducting regular pathological examinations, and enhancing treatment strategies for individuals infected with HR‐HPV types and multiple HPV subtypes (including HR subtypes) [46].

Several studies in women have demonstrated that HPV infection is age‐specific [47, 48]. However, accurate data regarding age‐specific HPV prevalence in men remain limited. In our study, young men under the age of 25 exhibited the highest infection rates, likely due to their early sexual activity, immature immune response, and increased susceptibility to HPV infection [49, 50]. Conversely, the elevated rates in older men (≥ 56) may be attributed to immunosenescence, reactivation of latent infections and reduced healthcare‐seeking behavior resulting in longer‐duration infections. Therefore, implementing a vaccination program before the onset of sexual activity could be an effective strategy for reducing HPV infection rates in this population.

Current HPV vaccination options available in China include bivalent, quadrivalent, and nonavalent formulations [51]. Studies have demonstrated a notable reduction in HPV infection rates following vaccination [52], with evidence showing that vaccination can reduce the infection rates of high‐risk HPV genotypes by approximately 90% among women [53]. Although data on male HPV vaccination are relatively limited, existing studies indicate that men also derive significant benefits from vaccination, resulting in reduced HPV infection rates and associated health complications. Additionally, vaccinating men contributes to decreased HPV transmission, thereby indirectly protecting unvaccinated individuals [54]. Based on our genotype distribution analysis, we estimate that the 9vHPV vaccine could theoretically prevent ~65% of LR‐HPV and 42% of HR‐HPV infections among males in Shantou. However, a considerable proportion (58%) of HR‐HPV genotypes—particularly prevalent types such as HPV51, HPV39, and HPV59—are not covered by current vaccines. This coverage gap carries significant public health implications, indicating that even with optimal vaccine implementation, a substantial burden of HR‐HPV infection would remain in the male population. Currently, there is a lack of relevant research on the awareness and acceptance of the HPV vaccine among the local population. Future studies should aim to gather this information to better inform targeted vaccination strategies.

Given that HPV vaccines are not yet approved for use in males in mainland China, alternative strategies should be considered. These include: (1) accelerating the approval process for male HPV vaccination, prioritizing high‐risk groups; (2) developing region‐specific, next‐generation vaccines that target prevalent genotypes not covered by current formulations; (3) implementing targeted screening programs for males with high‐risk sexual behaviors; and (4) enhancing public education regarding HPV transmission and prevention. A cost‐effectiveness analysis of these interventions would provide valuable insights for shaping regional public health policy, especially in light of the high prevalence of nonvaccine‐covered HR‐HPV genotypes in this population.

This study has several limitations. First, as a cross‐sectional retrospective observational study, it is limited in its ability to establish causal relationships or track changes in HPV prevalence over time. A longitudinal design would be more appropriate for capturing temporal trends and identifying potential causal factors. Second, the relatively small sample size may not fully represent the broader male population in Shantou, highlighting the need for larger, more diverse studies. Third, the sample included a heterogeneous group of symptomatic and asymptomatic outpatients, as well as individuals with various sexually transmitted infections, which may not accurately reflect the HPV prevalence in healthy males. More targeted studies focused on the general male population are needed for more precise estimates. Additionally, due to limitations in the available medical records, we were unable to assess other key risk factors, such as the number of sexual behaviors, sexual partners, or socioeconomic status, all of which are crucial for understanding and managing HPV risk. Finally, the study did not assess the persistence or clearance of HPV infections, which are essential for understanding the natural history of HPV in men. Future research should address these gaps to improve our understanding of HPV epidemiology in the male population.

## 5 Conclusion

This study demonstrated a 44.27% HPV prevalence among Shantou men, with predominance of HR‐HPV types (HPV16, 52, 51) and LR‐HPV types (HPV6, 11). Based on these findings and the bimodal age distribution with peaks in younger (≤ 25) and older (≥ 56) men, we recommend: (1) expediting approval of male HPV vaccination in China, prioritizing adolescents before sexual debut; (2) developing region‐specific vaccines incorporating prevalent genotypes not covered by the 9vHPV vaccine, which would leave 58% of HR infections unaddressed; and (3) implementing age‐specific education campaigns and surveillance systems to monitor genotype distribution changes over time. These targeted interventions would more effectively reduce the burden of HPV infection among males in this region.

## Author Contributions

**Chusheng Huang:** conceptualization, data curation. **Tongtong Xiao:** conceptualization, data curation, investigation, methodology, visualization, writing – original draft. **Fan Yang:** data curation, writing – review and editing. **Xiaoxia Ma:** data curation, supervision, writing – original draft. All authors read and approved the final manuscript. All authors have read and approved the final version of the manuscript [CORRESPONDING AUTHOR or MANUSCRIPT GUARANTOR] had full access to all of the data in this study and takes complete responsibility for the integrity of the data and the accuracy of the data analysis.

## Ethics Statement

This study was approved by the Medical Ethics Committee of Shantou Central Hospital.

## Consent

As this study was a retrospective study and the identities of patients were intentionally anonymized, no individual informed consent was required, and subject informed consent was exempted.

## Conflicts of Interest

The authors declare no conflicts of interest.

## Transparency Statement

The lead author Tongtong Xiao affirms that this manuscript is an honest, accurate, and transparent account of the study being reported; that no important aspects of the study have been omitted; and that any discrepancies from the study as planned (and, if relevant, registered) have been explained.

## Data Availability

The data sets generated and/or analyzed during the current study are not publicly available due to the lack of an online platform, but are available from the corresponding author on reasonable request. The authors take full responsibility for the integrity of the data and the accuracy of the analysis.
